# Sample-size determination for the Bayesian *t* test and Welch’s test using the approximate adjusted fractional Bayes factor

**DOI:** 10.3758/s13428-020-01408-1

**Published:** 2020-07-06

**Authors:** Qianrao Fu, Herbert Hoijtink, Mirjam Moerbeek

**Affiliations:** Department of Methodology and Statistics, Utrecht University, PO Box 80140, 3508 TC, Utrecht, The Netherlands

**Keywords:** Bayes factor, Bayesian *t* test, Bayesian Welch’s test, Sample-size determination, SSDbain

## Abstract

When two independent means *μ*_1_ and *μ*_2_ are compared, *H*_0_ : *μ*_1_ = *μ*_2_, *H*_1_ : *μ*_1_≠*μ*_2_, and *H*_2_ : *μ*_1_ > *μ*_2_ are the hypotheses of interest. This paper introduces the R package SSDbain, which can be used to determine the sample size needed to evaluate these hypotheses using the approximate adjusted fractional Bayes factor (AAFBF) implemented in the R package bain. Both the Bayesian *t* test and the Bayesian Welch’s test are available in this R package. The sample size required will be calculated such that the probability that the Bayes factor is larger than a threshold value is at least *η* if either the null or alternative hypothesis is true. Using the R package SSDbain and/or the tables provided in this paper, psychological researchers can easily determine the required sample size for their experiments.

## Introduction

In the Neyman–Pearson approach to hypothesis testing (Gigerenzer, 2004) a null and an alternative hypothesis are compared. Suppose the population means of males and females are denoted by *μ*_1_ and *μ*_2_. Three hypotheses are relevant: the null hypothesis *H*_0_: *μ*_1_ = *μ*_2_, the two-sided alternative hypothesis *H*_1_: *μ*_1_≠*μ*_2_, and the one-sided alternative hypothesis *H*_2_: *μ*_1_ > *μ*_2_. The null hypothesis *H*_0_ is rejected if the observed absolute *t*-statistic falls inside the critical region, where the critical region is a set of values that are equal to or greater than the critical value *t*_1−*α*/2,*v*_, where *α* is the type I error rate, and *v* is the degree of freedom for a two-sided alternative hypothesis. The null hypothesis *H*_0_ is rejected if the observed *t*-statistic falls inside the critical region, where the critical region is a set of values that are equal to or greater than the critical value *t*_1−*α*,*v*_ for a one-sided alternative hypothesis (Gigerenzer 1993, 2004). Statistical power is the probability of finding an effect when it exists in the population, that is, the probability of rejecting the null hypothesis when the alternative is true. Power analysis for Neyman–Pearson hypothesis testing has been studied for more than 50 years. Cohen (1992, 1988) played a pioneering role in the development of effect sizes and power analysis, and he provided mathematical equations for the relation between effect size, sample size, type I error rate and power. For example, if one aims for a power of 80%, the minimum sample size per group should be 394, 64 and 26 for small (*d* = 0.2), medium (*d* = 0.5) and large (*d* = 0.8) effect sizes, respectively for an independent samples two-sided *t* test at type I error rate *α* = .05, where Cohen’s *d* is the standardized difference between two means. To perform statistical power analyses for various tests, the G⋆Power program was developed by Erdfelder et al., (1996) and Faul et al., (2007) and Mayr et al., (2007). Despite the availability of G⋆Power there is still a lot of underpowered research in the behavioral and social sciences, even though criticism with respect to insufficient power is steadily increasing (Maxwell, 2004; Button et al., 2013; Simonsohn et al., 2014).

Numerous articles have criticized the Neyman–Pearson approach to hypothesis testing in the classical framework (e.g., Cohen (1994), Nickerson (2000), Sellke et al., (2001), Wagenmakers (2007), and Hubbard and Lindsay (2008)). As an alternative, Jeffreys (1961) and Kass and Raftery (1995) introduced the Bayes factor (BF). BF quantifies the relative support in the data for one hypothesis against another, and in addition to that, cannot only provide evidence in favor of the alternative hypothesis, but also provides evidence in favor of the null hypothesis. This approach for Bayesian hypothesis evaluation is increasingly receiving attention from psychological researchers, see for example Van de Schoot et al., (2017) and Vandekerckhove et al., (2018), and Wagenmakers et al., (2016). Nevertheless, researchers, especially psychologists, find it difficult to calculate BF and several software packages for Bayesian hypothesis evaluation have been developed. The most important are the R package BayesFactor (Rouder et al., 2009), that can be found at http://bayesfactorpcl.r-forge.r-project.org/ and the R package bain (Gu et al., 2018) that can be found at https://informative-hypotheses.sites.uu.nl/software/bain/. The latter is the successor of the stand-alone software BIEMS (Mulder et al., 2012) that can be found at https://informative-hypotheses.sites.uu.nl/software/biems/. Both BayesFactor and bain are implemented in JASP (https://jasp-stats.org/). The main difference between approximate adjusted fractional Bayes factor (AAFBF) implemented in bain and the Jeffreys–Zellner–Siow Bayes factor implemented in BayesFactor is the choice of the prior distribution. We focus on the AAFBF (to be elaborated in the next section) in this manuscript because it is available for both the *t* test and the Welch’s test.

When two independent group means are compared, there exist two specific cases in which variances are either equal or unequal for the two groups, which correspond to *t* test or Welch’s test. The *t* test is well known, while Welch’s test is often extremely important and useful as demonstrated by Ruscio and Roche (2012) and Rosopa et al., (2013), and Delacre et al., (2017). In the Neyman–Pearson approach to hypothesis testing, the formulae for calculating the sample size are given by an a priori power analysis for *t* test and Welch’s test (Cohen, 1992; Faul et al., 2007). There is not yet a solid body of literature regarding sample-size determination (SSD) for Bayesian hypothesis evaluation, but Weiss (1997) and De Santis (2004) and De Santis (2007) give different sample-size determination approaches for testing one mean of the normal distribution with known variance. Kruschke (2013) and Kruschke and Liddell (2018) discuss parameter estimation and use the posterior distribution as a measure of evidence strength, and Schönbrodt and Wagenmakers (2018) and Stefan et al., (2019) introduce Bayes factor design analysis applied to fixed-N and sequential designs. This paper will elaborate on these approaches in the following manners. (1) in addition to the Bayesian *t* test the Bayesian Welch’s test also will be considered. In practice, Welch’s test is more widely used, which is a necessary improvement in this manuscript; (2) both two-sided and one-sided alternative hypotheses are considered. One-sided alternative hypothesis can effectively reduce the required sample size and it is recommended to be used. This manuscript will provide a comprehensive analysis for both two-sided and one-sided alternative hypotheses; (3) the sample size will be calculated such that the probability that the Bayes factor is larger than a user specified threshold is at least *η* if either the null hypothesis or the alternative hypothesis is true; (4) we use the dichotomy method to compute the sample size very fast. In the previous publication, the sample size is computed through progressively increase the sample size with one until the threshold value is reached. This method is simple and easily used but with high computation effort, especially for the case when the required sample size is large, e.g., the sample size of 500 will cause several hundreds of iterations, while only 12 iterations are required with our method; (5) the sensitivity of SSD with respect to the specification of the prior will be highlighted. This is very important when Bayes factor is used for the hypothesis testing evaluation, because there exists some uncertainty for the required sample size for different prior distributions.

The outline of this paper is as follows. First, we give a brief introduction of the AAFBF, show how it can be computed, discuss the specification of the prior distribution and sensitivity analyses. Subsequently, sample-size determination is introduced. Thereafter, we will discuss the role of sample-size determination in Bayesian inference. The paper continues with an introduction of the ingredients required for sample-size determination. Then, the algorithm used to determine the sample size will be elaborated. Next, features of SSD are described. Thereafter, three examples are presented that will help psychological researchers to use the R package SSDbain if they plan to compare two independent means using the *t* test or the Welch’s test. The paper ends with a short conclusion.

## Bayes factor

In this paper, the means of two groups, *μ*_1_ and *μ*_2_, are compared for both Model 1: the within-group variances for group 1 and 2 are equal,

1 $$ y_{p} = \mu_{1} D_{1p}+ \mu_{2} D_{2p} + \epsilon_{p}~\text{with}~\epsilon_{p}\sim N(0,\sigma^{2}), $$

and Model 2: the within-group variances for group 1 and 2 are not equal,

2 $$ y_{p} = \mu_{1} D_{1p}+ \mu_{2} D_{2p} + \epsilon_{p}~\text{with}~\epsilon_{p}\sim N(0,D_{1p} {\sigma_{1}^{2}}+D_{2p} {\sigma_{2}^{2}}), $$

where *D*_1*p*_ = 1 for person *p* = 1,⋯ ,*N* and 0 otherwise, *D*_2*p*_ = 1 for person *p* = *N* + 1,⋯ ,2*N* and 0 otherwise, *N* denotes the common sample size for group 1 and 2, *𝜖*_*p*_ denotes the error in prediction, *σ*^2^ denotes the common within-group variance for group 1 and 2, and ${{\sigma _{1}^{2}}}$ and ${{\sigma _{2}^{2}}}$ denote the different within-group variances for group 1 and 2, respectively.

In this paper, the AAFBF (Gu et al., 2018; Hoijtink et al., 2019) is used to test hypotheses: *H*_0_ : *μ*_1_ = *μ*_2_ against *H*_1_: *μ*_1_≠*μ*_2_^1^ or against *H*_2_ : *μ*_1_ > *μ*_2_. The Bayes factor (BF) quantifies the relative support in the data for a pair of competing hypotheses. Specifically, if BF_01_ = 5, the support in the data is five times stronger for *H*_0_ than for *H*_1_; if BF_01_ = 0.2, the support in the data is five times stronger for *H*_1_ than for *H*_0_. As was shown in Klugkist et al., (2005) the BF in terms of comparing the constrained hypothesis *H*_*i*_ (*i* = 0,2) with the hypothesis *H*_1_ can be expressed in a simple form:

3 $$ \text{BF}_{i1} = \frac{f_{i}}{c_{i}}, $$

where *c*_*i*_ denotes the complexity of the hypothesis *H*_*i*_, and *f*_*i*_ denotes the fit of the hypothesis *H*_*i*_. The complexity *c*_*i*_ (a hypothesis with smaller complexity provides more precise predictions) of *H*_*i*_ describes how specific *H*_*i*_ is, and the corresponding fit *f*_*i*_ (the higher the fit the more a hypothesis is supported by the data) describes how well the data support *H*_*i*_. The formulae of the fit and complexity are:

4 $$ f_{i} = {\int}_{\boldsymbol \mu \in H_{i}} g_{1}(\boldsymbol \mu\mid \boldsymbol y, \boldsymbol D_{1}, \boldsymbol D_{2}) d\boldsymbol \mu, $$

5 $$ c_{i} = {\int}_{\boldsymbol \mu \in H_{i}} h_{1}(\boldsymbol \mu \mid \boldsymbol y, \boldsymbol D_{1}, \boldsymbol D_{2}) d\boldsymbol \mu, $$

where *g*_1_ (***μ***∣***y***,***D***_1_,***D***_2_) denotes the posterior distribution, and *h*_1_ (***μ***∣***y***,***D***_1_,***D***_2_) the prior distribution of ***μ*** under *H*_1_. In case of *H*_2_, *f*_2_ and *c*_2_ are the proportions of the posterior distribution *g*_1_(⋅) and prior distribution *h*_1_(⋅) in agreement with *H*_2_, respectively; in case of *H*_1_ Eq. 3 reduces to the Savage–Dickey density ratio (Dickey, 1971; Wetzels et al., 2010). The BF for *H*_0_ against *H*_2_ is:

6 $$ \text{BF}_{02}= \frac{\text{BF}_{01}}{\text{BF}_{21}}=\frac{\left.f_{0} \middle /c_{0}\right.}{\left.f_{2} \middle /c_{2}\right.}. $$

Actually, *g*_1_(⋅) is a normal approximation of the posterior distribution of *μ*_1_ and *μ*_2_:

7 $$ g_{1}(\boldsymbol \mu \mid \boldsymbol y, \boldsymbol D_{1}, \boldsymbol D_{2})= N \left( \left[\begin{array}{c} \hat{\mu}_{1}\\ \hat{\mu}_{2} \end{array}\right], \left[\begin{array}{cc} \hat{\sigma}^{2}/N & 0\\ 0 & \hat{\sigma}^{2}/N \end{array}\right] \right), $$

when Model 1 is considered; and

8 $$ g_{1}(\boldsymbol \mu \mid \boldsymbol y, \boldsymbol D_{1}, \boldsymbol D_{2})= N \left( \left[\begin{array}{c} \hat{\mu}_{1}\\ \hat{\mu}_{2} \end{array}\right], \left[\begin{array}{cc} \hat{\sigma}_{1}^{2}/N & 0\\ 0 & \hat{\sigma}_{2}^{2}/N \end{array}\right] \right), $$

when Model 2 is considered, where $\hat {\mu }_{1}$ and $\hat {\mu }_{2}$ denote the maximum likelihood estimates of the means of group 1 and group 2, respectively, and $\hat {\sigma }^{2}$, $\hat {\sigma }_{1}^{2}$ and $\hat {\sigma }_{2}^{2}$ denote unbiased estimates of the within-group variances. Due to the normal approximation, the general form of the AAFBF can be used to evaluate hypothesis evaluation in a wide range of statistical models such as structural equation modeling, logistic regression, multivariate regression, AN(C)OVA, etc. Therefore, it is currently the most versatile method for Bayesian hypotheses evaluation.

The prior distribution is based on the fractional Bayes factor approach (O’Hagan, 1995; Mulder, 2014). It is constructed using a fraction of information in the data. As elaborated in Gu et al., (2018) and Hoijtink et al., (2019) the prior distribution is given by:

9 $$ h_{1}(\boldsymbol \mu \mid \boldsymbol y, \boldsymbol D_{1}, \boldsymbol D_{2})=N \left( \left[\begin{array}{c} 0\\ 0 \end{array}\right], \left[\begin{array}{cc} \frac{1}{b}\frac{\hat{\sigma}^{2}}{N} & 0\\ 0 & \frac{1}{b}\frac{\hat{\sigma}^{2}}{N} \end{array}\right] \right), $$

where *b* is the fraction of information in the data used to specify the prior distribution, when Model 1 is considered, and

10 $$ h_{1}(\boldsymbol \mu \mid \boldsymbol y, \boldsymbol D_{1}, \boldsymbol D_{2})=N \left( \left[\begin{array}{c} 0\\ 0 \end{array}\right], \left[\begin{array}{cc} \frac{1}{b}\frac{\hat{\sigma}_{1}^{2}}{N} & 0\\ 0 & \frac{1}{b}\frac{\hat{\sigma}_{2}^{2}}{N} \end{array}\right] \right), $$

when Model 2 is considered.

The prior distribution is *NOT* used to represent the prior knowledge about the effect size under *H*_1_ or *H*_2_. The prior distribution is chosen such that a default Bayesian hypothesis evaluation of *H*_0_ vs *H*_*i*_ is obtained, that is, subjective input from the researcher is not needed. This is an advantage of default Bayesian hypothesis evaluation because the vast majority of researchers want to evaluate *H*_0_ vs *H*_1_ or *H*_0_ vs *H*_2_ and do not want to evaluate the corresponding prior distributions. The default value of *b* used for the Bayesian *t* test and Welch’s test equals $\frac {1}{2N}$. This choice is inspired by the minimal training sample idea (Berger and Pericchi 1996, 2004), that is, turn a noninformative prior into a proper prior using a small proportion of the information in the data. For our situation this is equivalent to using one half observation from group 1 and one half observation from group 2 is used, which is in total one observation. This makes sense because the focus is on one contrast, that is, *μ*_1_ − *μ*_2_, which means that one parameter needs to be estimated. This choice is too some extend arbitrary, for example, we could also use 2*b* (one person is needed to estimate each mean) or 3*b* (one person for each mean and the half for the residual variance), which still maintains the spirit of the minimal training sample approach. In summary, the goal is to compare *H*_0_ with *H*_*i*_ (*i* = 1,2) by means of Bayes factor, but not comparing the prior distribution of *H*_0_ with *H*_*i*_ (*i* = 1,2) through the Bayes factor. To achieve this, the prior distributions are calibrated such that *H*_0_ and *H*_*i*_ can be evaluated without requiring user input. However, there is some uncertainty in the calibrating, hence the AAFBF can be computed using the fractions *b*, 2*b*, and 3*b*, and the required sample sizes can be computed accordingly.

As an illustration, Tables 1 and 2 list the BF for the comparison of *H*_0_ with the two-sided alternative *H*_1_ and the one-sided alternative *H*_2_, respectively, when equal within-groups variances are considered (Model 1). From Table 1, we can see that when *H*_0_ is true (e.g., the entry with *b*), the support in the observed data is 13 times larger for *H*_0_ than for *H*_1_; when *H*_1_ is true, the support in the observed data is 22 (1/0.045) times larger for *H*_1_ than for *H*_0_. Table 2 shows that the data were nearly 18 times more likely to support *H*_0_ when *H*_0_ is true; the support in the data is more than 45 (1/0.022) times more likely to support *H*_2_ when *H*_2_ is true. Therefore, for the same sample size per group, it is much easier to get strong evidence for the one-sided than for the two-sided hypothesis (e.g., compare the corresponding shaded areas of the columns BF_01_ in Table 1 and BF_02_ in Table 2, BF_20_= 1/BF_02_ is larger than BF_10_= 1/BF_01_). The fit is higher for the true hypothesis (e.g., see column *f*_0_ in Table 1, *f*_0_ = 2.816 when *H*_0_ is true is larger than *f*_0_ = 0.009 when *H*_1_ is true). As can be seen in Tables 1 and 2 (bottom two panels) the BF is sensitive to the choice of the fraction. The complexity *c*_0_ becomes larger for *H*_0_ if the fraction increases (from 0.209 to 0.295, then to 0.362), while the complexity *c*_2_ is not affected by the fraction for *H*_2_ (0.5 for any value of fraction). This is because the complexity of a hypothesis specified using only inequality constraints is independent of the fraction, see Mulder (2014) for a proof. The corresponding BF for *H*_0_ becomes smaller (e.g., in the column BF_01_, BF decreases from 13.49 to 9.54, then to 7.79), and the BF for *H*_2_ does not change.

**Table 1:** Fit and complexity when *H*_0_ is true or *H*_1_ is true

		$\bar {y}_{1}$	$\bar {y}_{2}$	*s*^2^	*N*	*f*_0_	*c*_0_	BF_01_
*H*_0_	*b*	0	0	1	100	2.816	0.209	13.488
*H*_1_		0.5	0	1	100	0.009	0.209	
*H*_0_	2*b*	0	0	1	100	2.816	0.295	9.537
*H*_1_		0.5	0	1	100	0.009	0.295	
*H*_0_	3*b*	0	0	1	100	2.816	0.362	7.787
*H*_1_		0.5	0	1	100	0.009	0.362	

$\bar {y}_{1}$ and $\bar {y}_{2}$ are the sample means of the two groups, *s*^2^ is the sample variance of the two groups, *N* is the sample size per group

**Table 2:** Fit and complexity when *H*_0_ is true or *H*_2_ is true

		$\bar {y}_{1}$	$\bar {y}_{2}$	*s*^2^	*N*	*f*_0_	*c*_0_	*f*_2_	*c*_2_	BF_01_	BF_21_	BF_02_
*H*_0_	*b*	0	0	1	100	2.816	0.209	0.379	0.500	13.488	0.758	17.788
*H*_2_		0.5	0	1	100	0.009	0.209	1.000	0.500	0.045	1.999	
*H*_0_	2*b*	0	0	1	100	2.816	0.295	0.379	0.500	9.537	0.758	12.578
*H*_2_		0.5	0	1	100	0.009	0.295	1.000	0.500	0.032	1.999	
*H*_0_	3*b*	0	0	1	100	2.816	0.362	0.379	0.500	7.787	0.758	10.270
*H*_2_		0.5	0	1	100	0.009	0.362	1.000	0.500	0.026	1.999	

$\bar {y}_{1}$ and $\bar {y}_{2}$ are the sample means of the two groups, *s*^2^ is the sample variance of the two groups, *N* is the sample size per group

## Criteria for sample-size determination

For the Neyman–Pearson approach to hypothesis testing power analysis renders an indication of the sample sizes needed to reject the null-hypothesis with a pre-specified probability if it is not true. If the sample sizes are sufficiently large, under-powered studies can be avoided (Maxwell, 2004). A power analysis is conducted prior to a research study, and can be executed if three ingredients, type I error rate, type II error rate, and effect size are given. The main difficulty is getting an a priori educated guess of the true effect size. In practice, often one of two approaches to choose the effect size is used: use an estimate of the effect size based on similar studies in the literature, experts’ opinion or a pilot study (Sakaluk, 2016; Anderson et al., 2017); or, use the smallest effect size that is considered to be relevantly different from zero for the study at hand (Perugini et al., 2014). If the chosen effect size is smaller than the unknown true effect size, the sample sizes will be larger than necessary, which can be costly or unethical, and if the chosen effect size is larger than the unknown true effect size, the sample sizes will be too small and the resulting study will be underpowered.

**Fig. 1:**
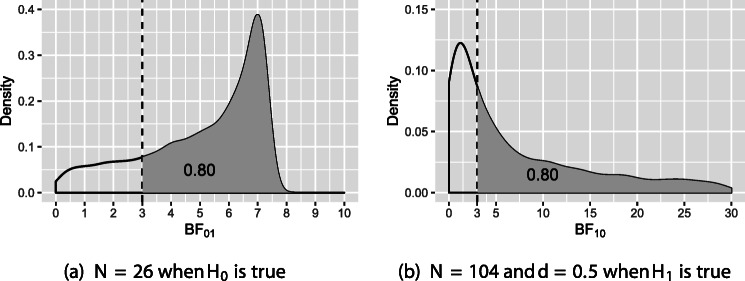
The sampling distribution of BF_01_ under *H*_0_ and BF_10_ under *H*_1_. The *vertical dashed line* denotes the BF_*thresh*_ = 3. The *grey area* visualizes *η* = 0.80. Note that, as will be illustrated in Table 4 later in this paper, the sample size is the maximum of 26 and 104

When the Bayes factor is used for hypothesis testing, sample-size determination instead of power analysis is used although the goals are similar. The main ingredients for SSD in a Bayesian framework are explained in Fig. 1. Panel (a) on the left: *t* test, sample size *N* = 26 per group, distribution of BF_01_ when data are repeatedly sampled from a population in which *H*_0_ : *μ*_1_ = *μ*_2_ is true. Panel (b) on the right: *t* test, sample size *N* = 104 per group, distribution of BF_10_ when data are repeatedly sampled from a population in which *μ*_1_≠*μ*_2_, but with the addition that the effect size has to be chosen (here we use effect size *d* = 0.5 to simulate data). We face the same problem as for power analysis, namely an unknown true effect size, but as will be elaborated in the next section, the combination of SSD and Bayesian updating can be used to address this problem.

Sample size will be determined such that *P*(BF_01_ > BF_*thresh*_|*H*_0_) ≥ *η* and *P*(BF_10_ > BF_*thresh*_|*H*_1_) ≥ *η*, that is, the probability that BF_01_ is larger than a user specified threshold value if *H*_0_ is true should be at least *η*, and the probability that BF_10_ is larger than the threshold value if *H*_1_ is true should be at least *η*. This is in line with power analysis in Neyman–Pearson approach to hypothesis testing in which the type I error rate *α* and type II error rate *β* are given beforehand. In the Bayesian framework, instead of type I error rate and type II error rates, we use the probability that the Bayes factor is larger than BF_*thresh*_ under the null hypothesis and under the alternative hypothesis. With respect to the choice of BF_*thresh*_, two situations can be distinguished. *Situation 1*: if one wants to explore which hypothesis is more likely to be supported, one can set BF_*thresh*_= 1. *Situation 2*: if one wants to find compelling evidence to support the true hypothesis, one can set BF_*thresh*_ equal to 3, 5, or 10, depending on the strength of the evidence that is required. With respect to the choice of *η* it should be noted that 1 − *η* are, for the null and alternative hypotheses, the Bayesian counterparts of the type I and the type II error rates. In high-stakes research, the probability of an erroneous decision should be small, therefore a larger value of *η* such as 0.90 should be used. In low-stakes or more exploratory research erroneous decisions may be less costly and smaller values like *η* = 0.80 could be used.

## The role of sample-size determination in Bayesian inference

In the Bayesian framework, updating (Rouder, 2014; Schönbrodt et al., 2017; Schönbrodt and Wagenmakers, 2018) can be seen as an alternative for sample-size determination that does not require specification of the effect size under the alternative hypothesis. Bayesian updating proceeds along the following steps: (i) specify an initial sample size per group and the required support in terms of BF; (ii) collect data with the initial sample size; (iii) compute the BF; (iv) if the support in favor of either *H*_0_ or *H*_1_ is large enough the study is finished; if the support is not large enough, increase the sample size and return to (iii). Because in the Bayesian framework the goal is not to control the Type I and Type II error rates (the goal is to quantify the support in the data for the hypotheses under consideration) this is a valid procedure.

With the availability of Bayesian updating and sample-size determination, two strategies can be used to obtain sufficient support for the hypotheses under consideration, which will be described in the next two sub-sections: (i) sample-size determination as a pre-experimental phase in case updating is not an option; and, (ii) sample-size determination followed by updating.

### Sample-size determination as a pre-experimental phase

If updating can be used, it is an approach that avoids pre-specification of the effect size under the alternative hypothesis and is a worthwhile option to pursue. However, updating cannot always be used or sample-size determination is a required step before updating can be executed. Consider the following situations. *Situation 1.* The population of interest is small, for instance, persons with a rare disease or cognitive disorder. The control and treatment groups will very likely not be large. Updating is in this situation not an option. However, if, for example, a researcher is interested to detect an effect size of Cohen’s *d* (for the *t* test) equal to .8 with a probability *η* = 0.80 that the Bayes factor is at least 5, the sample size required is 67 per group (see Table 5, which will be discussed after the next two sections). Since such a large sample size cannot be obtained, it is decided not to execute the experiment in this form. *Situation 2.* Next month a survey will start in which 150, currently single, men and women will be tracked for 21 years. Updating is not an option in such a longitudinal cohort study, but Table 4 shows that 104 persons per group are needed to have a probability of at least *η* = 0.80 to obtain a Bayes factor larger than 3 if the effect size is Cohen’s *d* = .5. Since the effect size is expected to be 0.5, the study can be actually conducted because the sample size is 150 persons per group. *Situation 3.* The researchers have to submit the research plans to the (medical) ethical committee. They want to use updating, but both the researchers and the committee’s members may want an indication of the sample size needed to obtain sufficient support for different effect sizes under the alternative hypothesis. Only with these numbers they can argue that they have sufficient funding and research time to execute the research plan. Sample-size determination can be used to obtain an indication of the sample sizes needed to obtain sufficient support for different effect sizes. These numbers can be included in the researcher’s research proposal for the (medical) ethical committee.

### Sample-size determination followed by updating

When sample-size determination is used, however, as will be highlighted using Situations 4 and 5, having to specify the effect size under the alternative hypothesis may have two undesirable consequences. Consider the following situations. *Situation 4.* If the alternative hypothesis is true, the researchers expect an effect size Cohen’s *d* = .5. They determine the sample sizes such that an effect size of Cohen’s *d* (for the *t* test) equal to .5 with *η* = 0.80 that the Bayes factor is at least 3 is detected, that is, 104 persons per group. After collecting data, they obtain BF_01_ = 2.5. This is an undesirable result because they did not achieve the desired support. They can remedy this by updating, that is, increasing the sample size until the Bayes factor is at least 3. The latter is only possible if updating is an option. *Situations 1 and 2* are examples of cases where this is not an option. *Situation 5.* Analogous to *Situation 4*, but now the researchers find BF_01_ = 8.3. This is a problem in the sense that they spent more funds and research time than would have been necessary. The researchers plan and are able to collect the data from 104 persons per group. If the research design permits this they can update until they reach the required support (which may be achieved at a sample size smaller than 104 per group), which will save funds and research time. The combination of sample-size determination and updating is the most powerful approach, whenever it is applicable.

## Ingredients for sample-size determination

Sample-size determination for the Bayesian *t* test and the Bayesian Welch’s test is implemented in the function SSDttest of the R package SSDbain available at https://github.com/Qianrao-Fu/SSDbain. In this section, we introduce and discuss the necessary input for sample-size determination with the SSDttest function. In the sections that follow, we will provide the algorithms used for Bayesian SSD, and a discussion of SSD properties using three tables for Cohen’s *d* equal to .2, .5, and .8, respectively. Furthermore, three examples of the application of SSDttest are presented.

After loading the SSDbain library, the following call is used to determine the sample size per group:

library(SSDbain)SSDttest(type='equal',Population_mean=c(0.5,0),var=NULL,BFthresh=3,eta=0.80,Hypothesis ='two-sided',T=10000)

The following ingredients are used:

1. type, a string that specifies the type of the test. If type='equal', the *t* test is used; if type='unequal', the Welch’s test is used. The default setting is type='equal'. If one expects (based on prior knowledge or prior evidence) that the two within-group variances are equal, choose the Bayesian *t* test, otherwise, choose the Bayesian Welch’s test (Ruxton, 2006; Ruscio & Roche, 2012; Delacre et al., 2017).
2. Population_mean, vector of length 2 specifying the population means of the two groups under *H*_1_ or *H*_2_. The default setting is Population_mean=c(0.5,0) when the effect size is *d* = 0.5. Note that, if var=NULL and the population mean in group 2 equals 0, the population mean in group 1 is identical to Cohen’s *d*.
3. var, vector of length 2 giving the two within-group variances. If type='equal', the default is var=c(1,1); if type='unequal', the default is var=c(4/3,2/3). Of course, any values of the variances can be used as input for the argument var.
4. BFthresh, a numeric value that specifies the magnitude of Bayes factor, e.g., 1, 3, 5, 10. The default setting is BFthresh= 3. If one chooses 5, one requires that BF_01_ is at least 5 if the data comes from a population in which *H*_0_ is true, and the BF_10_ is at least 5 if the data comes from a population in which *H*_1_ or *H*_2_ is true. The choice for the BFthresh value is subjective meaning that different values may be chosen by different researchers, for different studies and in different fields of science. A large BFthresh value may be chosen in high-stakes research were the degree of support of a hypothesis against another needs to be large. In pharmaceutical research for instance, the chances to have a new drug for cancer to be approved may be larger if there is high support for it increasing life expectancy as compared to an existing drug, especially so when the new drug may have side effects. A lower BFthresh value may be chosen in low-stakes research. An example also comes from pharmaceutical research, where a new headache relief drug and an existing competitor are compared on their onset of action, and side effects are not likely to occur.
5. eta, a numeric value that specifies the probability that the Bayes factor is larger than the BFthresh if either the null hypothesis or the alternative hypothesis is true, e.g., 0.80, 0.90. The default setting is eta= 0.80.
6. Hypothesis, a string that specifies the hypothesis. Hypothesis='two-sided' when the competing hypotheses are *H*_0_ : *μ*_1_ = *μ*_2_, *H*_1_ : *μ*_1_≠*μ*_2_; Hypothesis='one-sided' when the competing hypotheses are *H*_0_ : *μ*_1_ = *μ*_2_, *H*_2_ : *μ*_1_ > *μ*_2_. The default setting is Hypothesis='two-sided'. This argument is used to decide whether a two-sided (labelled *H*_1_ earlier in the paper) or a one-sided (labeled *H*_2_ earlier in the paper) alternative hypothesis is to be used. For example, one may wish to compare a new drug with an existing drug. If the researcher is not certain if the new drug will be more or less effective than the existing drug, a two-sided alternative hypothesis should be chosen. If the researcher has strong reasons to believe the new drug is more effective than the old one, a one-sided alternative hypothesis should be chosen.
7. T, a positive integer that specifies the number of data sets sampled from the null and alternative populations to determine the required sample size. The default setting is T = 10,000, and the recommended value is at least 10,000. This argument will be elaborated in the next section.

The output results include the sample size required and the corresponding probability that the Bayes factor is larger than the BF_*thresh*_ when either the null hypothesis or the alternative hypothesis is true:

Using N=xxx and bP(BF0i>BFthresh|H0)=xxxP(BFi0>BFthres}|Hi)=xxxUsing N=xxx and 2bP(BF0i>BFthresh|H0)=xxxP(BFi0>BFthresh|Hi)=xxxUsing N=xxx and 3bP(BF0i>BFthresh|H0)=xxxP(BFi0>BFthresh|Hi)=xxx

where xxx will be illustrated in the examples that will be given after the next section.

## Algorithm used in Bayesian sample-size determination

**Fig. 2:**
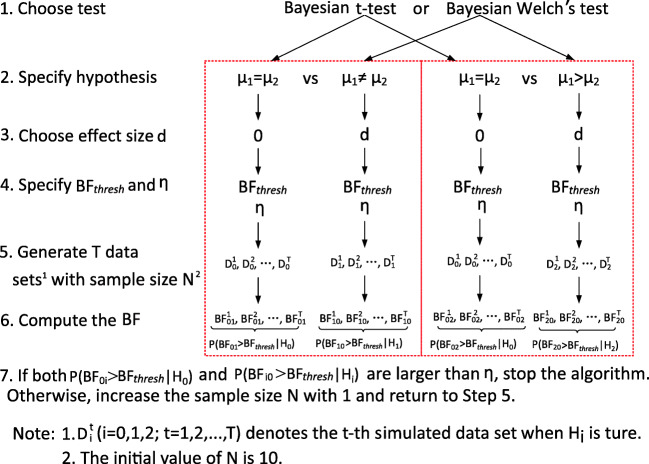
Algorithm 1: Sample-size determination for the Bayesian *t* test and Welch’s test

Figure 2 presents Algorithm 1, which is the basic algorithm used to determine the sample size. The ingredients in the first four Steps have been discussed in the previous section. In Step 5, *T* = 10,000 data sets are sampled from each of the populations of interest (e.g., *H*_0_ vs. *H*_1_), starting with a sample size *N* = 10 per group. In Step 6 the Bayes factor for each data set sampled from each hypothesis is computed. In Step 7, the probabilities *P*(BF_*i*1_ > BF_*thresh*_|*H*_0_) and *P*(BF_*i*0_ > BF_*thresh*_|*H*_*i*_) are computed. If both are larger than *η* specified in Step 4, the output presented in the previous section is provided. If one or both are smaller than *η*, *N* is increased by 1 per group and the algorithm restarts in Step 5. To be able to account for the sensitivity of the Bayes factor to the specification of the prior distribution, this algorithm is executed using fractions equal to *b*, 2*b*, and 3*b*. The A presents a refinement of Algorithm 1 that reduces the number of iterations in Algorithm 1 to maximally 12.

## Features of SSD

In this section, features of SSD will be discussed. This will be done using Tables 3, 4 and 5, which were constructed using SSDttest. The tables differ in effect size: Table 3 is for effect size *d* = 0.2, Table 4 is for effect size *d* = 0.5, and Table 5 is for effect size *d* = 0.8. The following features will be discussed: difference between the Bayesian *t* test and Bayesian Welch’s test, effect of the effect sizes, effect of the fraction *b* used to construct the prior distribution, and comparison of the two-sided and one-sided alternative hypothesis.

**Table 3:** When effect size *d* = 0.2, the sample size *N*, the corresponding probabilities *P*(BF_0*i*_ > BF_*thresh*_|*H*_0_) and *P*(BF_*i*0_ > BF_*thresh*_|*H*_*i*_) for the *t* test ^1^ and Welch’s test ^2^

			type	*t* test				Welch’s test			
			*η*	0.80		0.90		0.80		0.90	
			output	*N*	*P*(BF > BF_*thresh*_)	*N*	*P*(BF > BF_*thresh*_)	*N*	*P*(BF > BF_*thresh*_)	*N*	*P*(BF > BF_*thresh*_)
*b*	BF_*thresh*_ = 1	two-sided	*H*_0_	618	0.99	805	0.99	612	0.99	798	0.99
			*H*_1_		0.81		0.90		0.80		0.90
		one-sided	*H*_0_	507	0.99	676	0.99	508	0.99	682	0.99
			*H*_2_		0.80		0.90		0.80		0.90
	BF_*thresh*_ = 3	two-sided	*H*_0_	769	0.98	985	0.98	769	0.98	985	0.98
			*H*_1_		0.80		0.90		0.81		0.91
		one-sided	*H*_0_	676	0.97	863	0.98	666	0.97	864	0.98
			*H*_2_		0.80		0.90		0.80		0.90
	BF_*thresh*_ = 5	two-sided	*H*_0_	842	0.96	1048	0.96	845	0.96	1048	0.96
			*H*_1_		0.80		0.90		0.80		0.90
		one-sided	*H*_0_	743	0.95	939	0.96	743	0.95	941	0.96
			*H*_2_		0.80		0.90		0.80		0.90
2*b*	BF_*thresh*_ = 1	two-sided	*H*_0_	559	0.99	749	0.99	564	0.99	749	0.99
			*H*_1_		0.80		0.90		0.81		0.90
		one-sided	*H*_0_	460	0.99	625	0.99	460	0.99	623	0.99
			*H*_2_		0.80		0.90		0.80		0.90
	BF_*thresh*_ = 3	two-sided	*H*_0_	722	0.96	913	0.97	722	0.96	926	0.97
			*H*_1_		0.80		0.90		0.80		0.91
		one-sided	*H*_0_	625	0.96	812	0.96	629	0.96	807	0.96
			*H*_2_		0.80		0.90		0.80		0.90
	BF_*thresh*_ = 5	two-sided	*H*_0_	805	0.94	998	0.95	799	0.94	997	0.94
			*H*_1_		0.81		0.90		0.80		0.90
		one-sided	*H*_0_	699	0.92	890	0.93	699	0.92	886	0.93
			*H*_2_		0.80		0.90		0.81		0.90
3*b*	BF_*thresh*_ = 1	two-sided	*H*_0_	534	0.99	699	0.99	526	0.99	699	0.99
			*H*_1_		0.80		0.90		0.80		0.90
		one-sided	*H*_0_	429	0.98	588	0.99	429	0.98	582	0.99
			*H*_2_		0.81		0.90		0.81		0.90
	BF_*thresh*_ = 3	two-sided	*H*_0_	699	0.95	889	0.96	704	0.95	889	0.96
			*H*_1_		0.81		0.90		0.81		0.90
		one-sided	*H*_0_	590	0.94	781	0.95	592	0.94	769	0.95
			*H*_2_		0.80		0.90		0.80		0.90
	BF_*thresh*_ = 5	two-sided	*H*_0_	765	0.92	967	0.93	767	0.91	967	0.93
			*H*_1_		0.80		0.90		0.80		0.90
		one-sided	*H*_0_	668	0.90	858	0.92	672	0.90	868	0.91
			*H*_2_		0.81		0.90		0.80		0.91

^1^ the means *μ*_1_ = 0.2, *μ*_2_ = 0 and the variance *σ*^2^ = 1

^2^ the means *μ*_1_ = 0.2, *μ*_2_ = 0 and the variances ${\sigma ^{2}_{1}}=1.33$, ${\sigma ^{2}_{2}}=0.67$

**Table 4:** When effect size *d* = 0.5, the sample size *N*, the corresponding probabilities *P*(BF_0*i*_ > BF_*thresh*_|*H*_0_) and *P*(BF_*i*0_ > BF_*thresh*_|*H*_*i*_) for the *t* test ^1^ and Welch’s test ^2^

			type	*t* test				Welch’s test			
			*η*	0.80		0.90		0.80		0.90	
			output	*N*	*P*(BF > BF_*thresh*_)	*N*	*P*(BF > BF_*thresh*_)	*N*	*P*(BF > BF_*thresh*_)	*N*	*P*(BF > BF_*thresh*_)
*b*	BF_*thresh*_ = 1	two-sided	*H*_0_	77	0.97	104	0.98	77	0.97	104	0.98
			*H*_1_		0.80		0.90		0.80		0.90
		one-sided	*H*_0_	59	0.97	84	0.97	59	0.97	84	0.97
			*H*_2_		0.80		0.90		0.80		0.90
	BF_*thresh*_ = 3	two-sided	*H*_0_	104	0.92	133	0.94	104	0.92	133	0.94
			*H*_1_		0.80		0.90		0.80		0.90
		one-sided	*H*_0_	87	0.91	115	0.92	87	0.91	115	0.92
			*H*_2_		0.81		0.90		0.81		0.90
	BF_*thresh*_ = 5	two-sided	*H*_0_	115	0.86	191	0.91	115	0.86	191	0.91
			*H*_1_		0.80		0.97		0.80		0.97
		one-sided	*H*_0_	99	0.84	207	0.90	100	0.84	209	0.90
			*H*_2_		0.80		0.99		0.81		0.99
2*b*	BF_*thresh*_ = 1	two-sided	*H*_0_	67	0.96	93	0.97	67	0.96	94	0.97
			*H*_1_		0.80		0.90		0.80		0.90
		one-sided	*H*_0_	49	0.95	73	0.96	49	0.95	73	0.96
			*H*_2_		0.80		0.90		0.80		0.90
	BF_*thresh*_ = 3	two-sided	*H*_0_	96	0.87	130	0.90	96	0.87	139	0.90
			*H*_1_		0.80		0.92		0.81		0.93
		one-sided	*H*_0_	79	0.85	158	0.90	79	0.85	156	0.90
			*H*_2_		0.81		0.98		0.80		0.98
	BF_*thresh*_ = 5	two-sided	*H*_0_	128	0.80	369	0.90	127	0.80	379	0.90
			*H*_1_		0.88		1.00		0.87		1.00
		one-sided	*H*_0_	134	0.81	420	0.90	134	0.80	422	0.90
			*H*_2_		0.93		1.00		0.93		1.00
3*b*	BF_*thresh*_ = 1	two-sided	*H*_0_	63	0.95	87	0.96	63	0.95	87	0.96
			*H*_1_		0.81		0.91		0.81		0.90
		one-sided	*H*_0_	43	0.92	67	0.94	43	0.92	67	0.94
			*H*_2_		0.81		0.91		0.81		0.90
	BF_*thresh*_ = 3	two-sided	*H*_0_	92	0.83	196	0.90	91	0.83	208	0.91
			*H*_1_		0.81		0.99		0.80		0.99
		one-sided	*H*_0_	74	0.81	230	0.90	74	0.81	226	0.90
			*H*_2_		0.81		1.00		0.81		1.00
	BF_*thresh*_ = 5	two-sided	*H*_0_	191	0.81	551	0.90	196	0.80	580	0.91
			*H*_1_		0.98		1.00		0.98		1.00
		one-sided	*H*_0_	199	0.80	608	0.90	200	0.80	622	0.90
			*H*_2_		0.99		1.00		0.99		1.00

^1^ the means *μ*_1_ = 0.5, *μ*_2_ = 0 and the variance *σ*^2^ = 1

^2^ the means *μ*_1_ = 0.5, *μ*_2_ = 0 and the variances ${\sigma ^{2}_{1}}=1.33$, ${\sigma ^{2}_{2}}=0.67$

**Table 5:** When effect size *d* = 0.8, the sample size *N*, the corresponding probabilities *P*(BF_0*i*_ > BF_*thresh*_|*H*_0_) and *P*(BF_*i*0_ > BF_*thresh*_|*H*_*i*_) for the *t* test ^1^ and Welch’s test ^2^

			type	*t* test				Welch’s test			
			*η*	0.80		0.90		0.80		0.90	
			output	*N*	*P*(BF > BF_*thresh*_)	*N*	*P*(BF > BF_*thresh*_)	*N*	*P*(BF > BF_*thresh*_)	*N*	*P*(BF > BF_*thresh*_)
*b*	BF_*thresh*_ = 1	two-sided	*H*_0_	25	0.95	36	0.96	26	0.95	35	0.96
			*H*_1_		0.80		0.91		0.81		0.90
		one-sided	*H*_0_	18	0.93	27	0.95	18	0.93	27	0.95
			*H*_2_		0.81		0.90		0.81		0.90
	BF_*thresh*_ = 3	two-sided	*H*_0_	36	0.85	72	0.90	36	0.85	73	0.90
			*H*_1_		0.80		0.98		0.80		0.98
		one-sided	*H*_0_	30	0.82	81	0.91	30	0.82	79	0.90
			*H*_2_		0.81		1.00		0.81		0.99
	BF_*thresh*_ = 5	two-sided	*H*_0_	67	0.80	191	0.91	66	0.80	191	0.91
			*H*_1_		0.96		1.00		0.96		1.00
		one-sided	*H*_0_	67	0.80	207	0.90	67	0.80	209	0.90
			*H*_2_		0.98		1.00		0.98		1.00
2*b*	BF_*thresh*_ = 1	two-sided	*H*_0_	21	0.91	31	0.93	21	0.91	31	0.93
			*H*_1_		0.80		0.90		0.80		0.90
		one-sided	*H*_0_	14	0.88	23	0.91	14	0.88	23	0.91
			*H*_2_		0.82		0.90		0.82		0.90
	BF_*thresh*_ = 3	two-sided	*H*_0_	48	0.80	130	0.90	48	0.80	139	0.90
			*H*_1_		0.93		1.00		0.92		1.00
		one-sided	*H*_0_	48	0.80	158	0.90	46	0.80	156	0.90
			*H*_2_		0.96		1.00		0.95		1.00
	BF_*thresh*_ = 5	two-sided	*H*_0_	128	0.80	369	0.90	127	0.80	379	0.90
			*H*_1_		1.00		1.00		1.00		1.00
		one-sided	*H*_0_	134	0.81	420	0.90	134	0.80	422	0.90
			*H*_2_		1.00		1.00		1.00		1.00
3*b*	BF_*thresh*_ = 1	two-sided	*H*_0_	19	0.88	29	0.91	19	0.87	29	0.91
			*H*_1_		0.80		0.91		0.81		0.91
		one-sided	*H*_0_	10	0.82	26	0.90	10	0.80	26	0.91
			*H*_2_		0.80		0.94		1.01		0.94
	BF_*thresh*_ = 3	two-sided	*H*_0_	73	0.81	196	0.90	73	0.80	208	0.91
			*H*_1_		0.99		1.00		0.99		1.00
		one-sided	*H*_0_	70	0.81	230	0.90	73	0.81	226	0.90
			*H*_2_		0.99		1.00		1.00		1.00
	BF_*thresh*_ = 5	two-sided	*H*_0_	191	0.81	551	0.90	196	0.80	580	0.91
			*H*_1_		1.00		1.00		1.00		1.00
		one-sided	*H*_0_	199	0.80	608	0.90	200	0.80	622	0.90
			*H*_2_		1.00		1.00		1.00		1.00

^1^ the means *μ*_1_ = 0.8, *μ*_2_ = 0 and the variance *σ*^2^ = 1

^2^ the means *μ*_1_ = 0.8, *μ*_2_ = 0 and the variances ${\sigma ^{2}_{1}}=1.33$, ${\sigma ^{2}_{2}}=0.67$

There seems to be little difference between the *t* test and Welch’s test with respect to the sample size required and the corresponding probability that the Bayes factor is larger than BF_*thresh*_ if either the null or the alternative hypothesis is true. For example, for BF_*thresh*_= 3, two-sided testing, effect size *d* = 0.5, and *η* = 0.80 (see Table 4), the sample size is 104 per group, and the probability that the Bayes factor is larger than 3 if *H*_0_ is true is 0.92, and the probability that the Bayes factor is larger than 3 if *H*_1_ is true is 0.80 for the *t* test. The sample size is 104 per group, and the probability that the Bayes factor is larger than 3 if *H*_0_ is true is 0.92, and the probability that the Bayes factor is larger than 3 if *H*_1_ is true is 0.80 for Welch’s test.

As expected, the sample size required decreases as the effect size under *H*_*i*_ increases. For example, for the two-sided *t* test, BF_*thresh*_= 3 and *η* = 0.80, the sample sizes required for effect sizes 0.2, 0.5, and 0.8 are 769, 104, and 36 per group, respectively. This is because an increase of the effect size makes the alternative more distinguishable from the null hypothesis. However, for some special cases, the sample size required for effect size 0.5 and 0.8 are the same, for example for the two-sided *t* test, BF_*thresh*_= 5 and *η* = 0.80 if the fraction 2*b* is used for the prior distribution. The reason is that the sample size required is the maximum of the sample size required if the null hypothesis is true and the sample size required if the alternative hypothesis is true. In cases like the examples given, the maximum sample size is determined by the null hypothesis, which is the same for effect size 0.5 and 0.8.

The sample size required increases with the fraction going from *b* to 2*b*, and then to 3*b* if the null hypothesis is true, while the opposite relation is found if the alternative hypothesis is true. This feature can be explained as follows: according to Equations (9) and (10), as the fraction gets larger, the prior variance decreases, the relative complexity *c*_0_ gets larger, thus the Bayes factor under *H*_0_ gets smaller. Consequently, the sample size required increases. Conversely, the sample size required when the alternative hypothesis is true decreases. This feature highlights that a sensitivity analysis is important: results depend on the fraction of information used to specify the prior distribution.

As can be seen in Tables 3–5, the required sample sizes for one-sided testing are always smaller than or about equal to the sample sizes required for two-sided testing. Therefore, if a directional hypothesis can be formulated, a one-sided testing is preferred over a two-sided testing.

## Practical examples of SSD

In this section, three examples of SSD will be given. The examples use the function SSDttest because it allows researchers to choose Cohen’s *d*, BF_*thresh*_, and *η* as they desire. As an alternative, researchers can also consult Tables 3, 4 and 5, although there sample sizes are only given for a limited number of values for Cohen’s *d*, BF_*thresh*_ and *η*.

### *Example 1*

Researchers want to conduct an experiment to investigate whether there is a difference in pain intensity as experienced by users of two types of local anesthesia. The researchers would like to detect a medium effect size *d* = 0.5 with a two-sided *t* test, when either *H*_0_ or *H*_1_ with *d* = 0.5 is true, such that they have a probability of 0.80 that the resulting Bayes factor is larger than 3. The researchers choose BF_*thresh*_ = 3 because they want to get a compelling evidence for the high-stakes experiment that one of the two types of anesthesia is better able to reduce the pain intensity for users. As elaborated below, the researchers can combine SSD with Bayesian updating to (i) stop sampling before a sample size of *N* = 104 per group if the true effect size is larger than *d* = 0.5 used for SSD, or (ii) to continue sampling beyond *N* = 104 per group if the true effect size is smaller than 0.50. The sample size required to detect *d* = 0.5 is obtained using the following call to SSDttest:

SSDttest(type='equal',Population_mean=c(0.5,0),var=c(1,1),BFthresh=3,eta=0.80,Hypothesis= 'two-sided',T=10000)

The results are as follows:

Using N=104 and bP(BF01>3|H0)=0.92P(BF10>3|H1)=0.80

The following can be learned from these results:

The researchers need to collect 104 cases per type of local anesthesia to get a probability of 0.92 that the resulting Bayes factor is larger than 3 when *H*_0_ is true, and to get a probability of 0.80 that the resulting Bayes factor is larger than 3 when *H*_1_ is true and *d* = 0.5.

The researchers will execute the Bayesian updating as follows. First, the researchers will start with 25% of the sample size per group, that is, 26 cases per group. If the resulting BF_01_ or BF_10_ is larger than 3, the desired support is achieved and updating can be stopped. Otherwise, the researchers can add 26 cases per group and recompute and re-evaluate the Bayes factors. Once the threshold of 3 has been achieved, this process can be stopped, otherwise it can be repeated, also beyond a sample size of 26 cases per group. The SSD executed before these researchers started collecting data is useful because it gives an indication of the sample size that are required to evaluated *H*_0_ and *H*_1_. Updating ensures that the researchers use their resources optimally.

### *Example 2*

Researchers want to carry out a test to explore whether there is a difference between the yield obtained with a new corn fertilizer and with a current fertilizer. They expect the new fertilizer is more effective than the current one. The researchers want to determine the number of field plots used in a study of the test to detect an effect size *d* = 0.2 with a one-sided *t* test. When either *H*_0_ or *H*_2_ with *d* = 0.2 is true they want to have a probability of 0.90 that the resulting Bayes factor is larger than 1. The researchers used BF_*thresh*_ = 1 and *η* = 0.90 because they want to get a Bayes factor to point to the true hypothesis with a high probability. They are not necessarily interested in strong evidence for the true hypothesis. The sample size required is obtained using the following call to SSDttest:

SSDttest(type='equal',Population_mean=c(0.2,0),var=c(1,1),BFthresh=1,eta=0.90,Hypothesis ='one-sided',T=10000)

The results are as follows:

Using N=676 and bP(BF02>1|H0)=0.99P(BF20>1|H2)=0.90

The following can be learned from the output:

The researchers need to collect 676 field plots per fertilizer to get a probability of 0.99 that the resulting Bayes factor is larger than 1 if *H*_0_ is true, and a probability of 0.90.16 that the resulting Bayes factor is larger than 1 if *H*_2_ is true.

### *Example 3*

Researchers wish to compare two weight loss regimens to determine whether there is a difference in the mean weight loss. Past experiments have shown that the standard deviations are different for these two regimens. Researchers want to determine the sample size required to detect the effect size *d* = 0.5 with a two-sided Welch’s test. When either *H*_0_ or *H*_1_ is true they want to have a probability of 0.80 that the resulting Bayes factor is larger than 3. They also want to execute a sensitivity analysis and therefore look at the sample sizes required for *b*, 2*b*, and 3*b*. The required sample size is obtained using the following call to SSDttest:

SSDttest(type='unequal',Population_mean=c(0.5,0),var=c(1.33,0.67),BFthresh=3,eta=0.80, Hypothesis='two-sided',T=10000)

The results are as follows:

Using N=104 and bP(BF01>3|H0)=0.92P(BF10>3|H1)=0.80Using N=96 and 2bP(BF01>3|H0)=0.87P(BF10>3|H1)=0.80Using N=91 and 3bP(BF01>3|H0)=0.83P(BF10>3|H1)=0.80

From the results the following can be learned:

The output from SSDttest can be used to perform a sensitivity analysis. As can be seen the required sample sizes for *b*, 2*b* and 3*b* are 104, 96, and 91 per group, respectively. This implies that if the researchers plan to execute a sensitivity analysis they should aim for a sample size of at least 104 per group. The probabilities of supporting *H*_0_ and *H*_1_ when they are true become more similar with bigger fractions of information. If this is a desirable feature for the researchers, they can use 3*b* which renders a required sample size of *N* = 91 per group and *η* is about equal to 0.80 both when *H*_0_ and *H*_1_ are true.

## Conclusions

The function SSDttest implemented in the R package SSDbain (https://github.com/Qianrao-Fu/SSDbain) has been developed for sample-size determination for two-sided and one-sided hypotheses under a Bayesian *t* test or Bayesian Welch’s test using the AAFBF as implemented in the R package bain. This function was used to construct sample size tables that are counterparts to the frequently used tables in Cohen (1992). If the tables are not applicable to the situation considered by researchers, the SSDbain package can be used.

With the growing popularity of Bayesian statistics (Van de Schoot et al., 2017), it is important tools for sample-size determination in the Bayesian framework become available. In this manuscript, we developed software to calculate sample sizes within the framework of Bayesian *t* test and Bayesian Welch’s test hypotheses using time-efficient algorithms. However, the SSDbain package also has its limitation: we focused on the AAFBF, but as was shortly highlighted in the introduction to this paper, there are other Bayes factors researchers may use. Furthermore, we focused on the Bayesian *t* test and Welch’s test, but in our future research we will extend to other statistical models, such as Bayesian ANOVA, ANCOVA, linear regression, and normal linear multivariate models.

## Appendix: : Algorithm 2

We have described the basic Algorithm 1 used to determine the sample size. In this appendix, a refinement of Algorithm 1 is described that reduces the number of iterations of Algorithm 1 to maximally 12. It is very time consuming to iterate Steps 5-7 many times in Algorithm 1, especially if the alternative hypothesis is one-sided. The number of iterations will be reduced if Step 7 from Algorithm 1 is replaced by Algorithm 2. The basic principle of Algorithm 2 is to gradually adjust the sample size using a dichotomy algorithm until *P*(BF_0*i*_ > BF_*t**h**r**e**s**h*_|*H*_0_) and *P*(BF_*i*0_ > BF_*t**h**r**e**s**h*_|*H*_*i*_) (*i* = 1, 2) hold for sample sizes ranging between $N_{\min \limits }=10$ and $N_{\max \limits }=1000$. If it turns out that $N_{\max \limits }$ is too small, its value will be increased. Using Algorithm 2 the number of iterations will be at most 12 ($O(\log _{2} (1000-10))+2= 12$) see https://en.wikipedia.org/wiki/Binary_search_algorithm for a detail.

1. If both *P*(BF_0*i*_ > BF_*t**h**r**e**s**h*_|*H*_0_) and *P*(BF_*i*0_ > BF_*t**h**r**e**s**h*_|*H*_*i*_) (*i* = 1, 2) are larger than *η*, set $N_{\max \limits }=N_{\text {mid}}$; otherwise, set $N_{\min \limits }=N_{\text {mid}}$, where $N_{\text {mid}}=(N_{\min \limits }+N_{\max \limits })/2$; and continue with (2).
2. If $N_{\text {mid}}=N_{\min \limits }+1$, then *N* = *N*_mid_, and the algorithm stops and output is provided; otherwise return to Step 5 from Algorithm 1 with *N* equal to *N*_mid_.

## Footnotes

1 Note that, *H*_1_ is equivalent to the unconstrained hypothesis *H*_*u*_ : *μ*_1_, *μ*_2_, in the sense that the Bayes factor for a constrained hypothesis versus *H*_1_ is the same as versus *H*_*u*_
